# Supplementary material for: Autophagy regulates lipid metabolism through selective turnover of NCoR1
Source: Nat Commun. 2019 Apr 5;10:1567. doi: 10.1038/s41467-019-08829-3 (PMC6450892; doi:10.1038/s41467-019-08829-3)
Supplement: Supplementary file 1 — Supplementary Information [file 41467_2019_8829_MOESM1_ESM.pdf]

Supplementary Information

Supplementary Figures

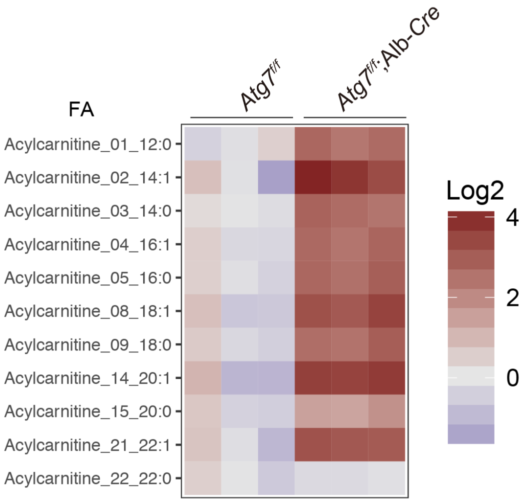

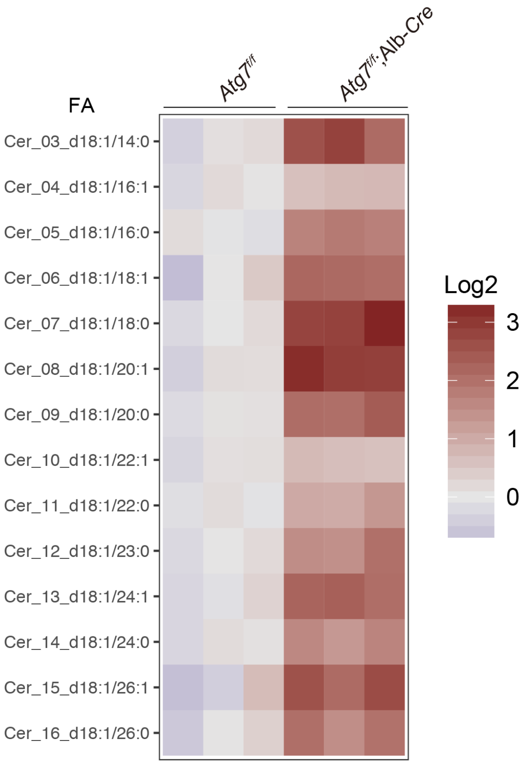

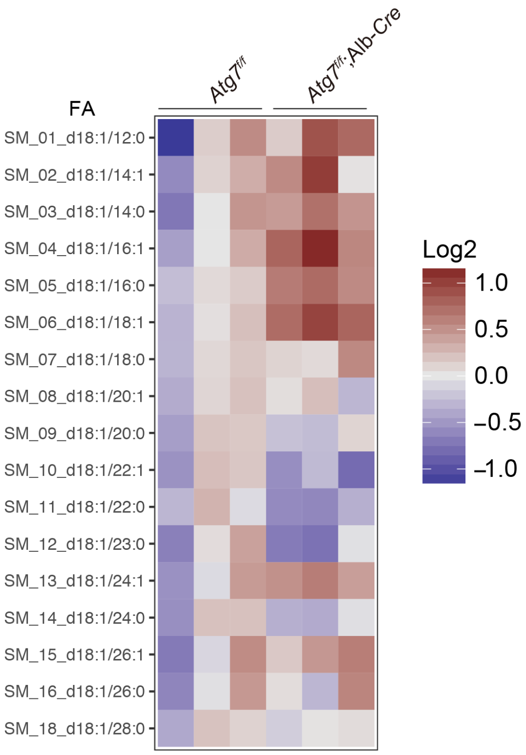

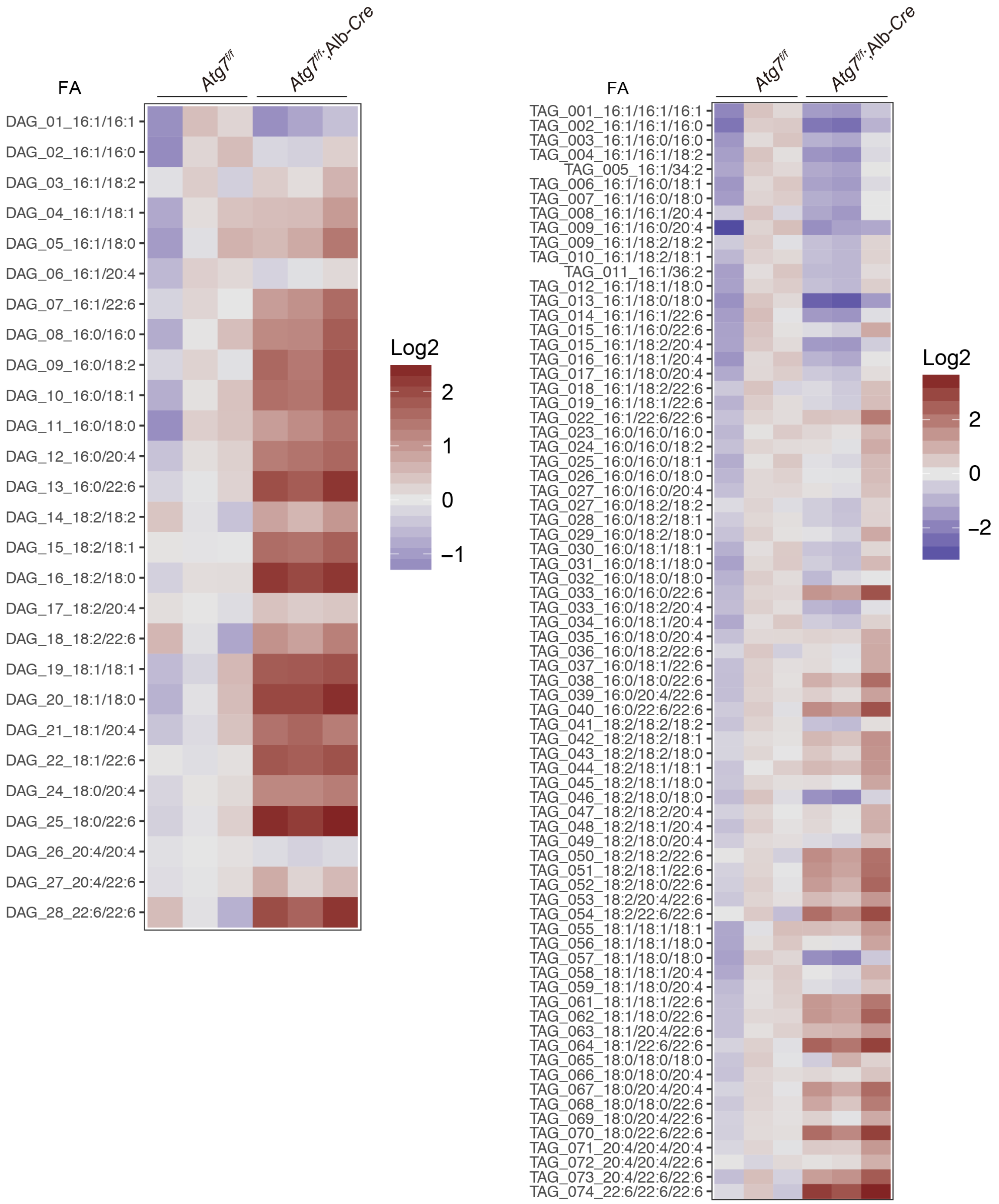
FA
Atg7f/f
Atg7f/f;Alb-Cre
DAG_01_16:1/16:1
DAG_02_16:1/16:0
DAG_03_16:1/18:2
DAG_04_16:1/18:1
DAG_05_16:1/18:0
DAG_06_16:1/20:4
DAG_07_16:1/22:6
DAG_08_16:0/16:0
DAG_09_16:0/18:2
DAG_10_16:0/18:1
DAG_11_16:0/18:0
DAG_12_16:0/20:4
DAG_13_16:0/22:6
DAG_14_18:2/18:2
DAG_15_18:2/18:1
DAG_16_18:2/18:0
DAG_17_18:2/20:4
DAG_18_18:2/22:6
DAG_19_18:1/18:1
DAG_20_18:1/18:0
DAG_21_18:1/20:4
DAG_22_18:1/22:6
DAG_24_18:0/20:4
DAG_25_18:0/22:6
DAG_26_20:4/20:4
DAG_27_20:4/22:6
DAG_28_22:6/22:6
Log2
2
1
0
−1
FA
Atg7f/f
Atg7f/f;Alb-Cre
TAG_001_16:1/16:1/16:1
TAG_002_16:1/16:1/16:0
TAG_003_16:1/16:0/16:0
TAG_004_16:1/16:1/18:2
TAG_005_16:1/34:2
TAG_006_16:1/16:0/18:1
TAG_007_16:1/16:0/18:0
TAG_008_16:1/16:1/20:4
TAG_009_16:1/16:0/20:4
TAG_009_16:1/18:2/18:2
TAG_010_16:1/18:2/18:1
TAG_011_16:1/36:2
TAG_012_16:1/18:1/18:0
TAG_013_16:1/18:0/18:0
TAG_014_16:1/16:1/22:6
TAG_015_16:1/16:0/22:6
TAG_015_16:1/18:2/20:4
TAG_016_16:1/18:1/20:4
TAG_017_16:1/18:0/20:4
TAG_018_16:1/18:2/22:6
TAG_019_16:1/18:1/22:6
TAG_022_16:1/22:6/22:6
TAG_023_16:0/16:0/16:0
TAG_024_16:0/16:0/18:2
TAG_025_16:0/16:0/18:1
TAG_026_16:0/16:0/18:0
TAG_027_16:0/16:0/20:4
TAG_027_16:0/18:2/18:2
TAG_028_16:0/18:2/18:1
TAG_029_16:0/18:2/18:0
TAG_030_16:0/18:1/18:1
TAG_031_16:0/18:1/18:0
TAG_032_16:0/18:0/18:0
TAG_033_16:0/16:0/22:6
TAG_033_16:0/18:2/20:4
TAG_034_16:0/18:1/20:4
TAG_035_16:0/18:0/20:4
TAG_036_16:0/18:2/22:6
TAG_037_16:0/18:1/22:6
TAG_038_16:0/18:0/22:6
TAG_039_16:0/20:4/22:6
TAG_040_16:0/22:6/22:6
TAG_041_18:2/18:2/18:2
TAG_042_18:2/18:2/18:1
TAG_043_18:2/18:2/18:0
TAG_044_18:2/18:1/18:1
TAG_045_18:2/18:1/18:0
TAG_046_18:2/18:0/18:0
TAG_047_18:2/18:2/20:4
TAG_048_18:2/18:1/20:4
TAG_049_18:2/18:0/20:4
TAG_050_18:2/18:2/22:6
TAG_051_18:2/18:1/22:6
TAG_052_18:2/18:0/22:6
TAG_053_18:2/20:4/22:6
TAG_054_18:2/22:6/22:6
TAG_055_18:1/18:1/18:1
TAG_056_18:1/18:1/18:0
TAG_057_18:1/18:0/18:0
TAG_058_18:1/18:1/20:4
TAG_059_18:1/18:0/20:4
TAG_061_18:1/18:1/22:6
TAG_062_18:1/18:0/22:6
TAG_063_18:1/20:4/22:6
TAG_064_18:1/22:6/22:6
TAG_065_18:0/18:0/18:0
TAG_066_18:0/18:0/20:4
TAG_067_18:0/20:4/20:4
TAG_068_18:0/18:0/22:6
TAG_069_18:0/20:4/22:6
TAG_070_18:0/22:6/22:6
TAG_071_20:4/20:4/20:4
TAG_072_20:4/20:4/22:6
TAG_073_20:4/22:6/22:6
TAG_074_22:6/22:6/22:6
Log2
2
0
−2

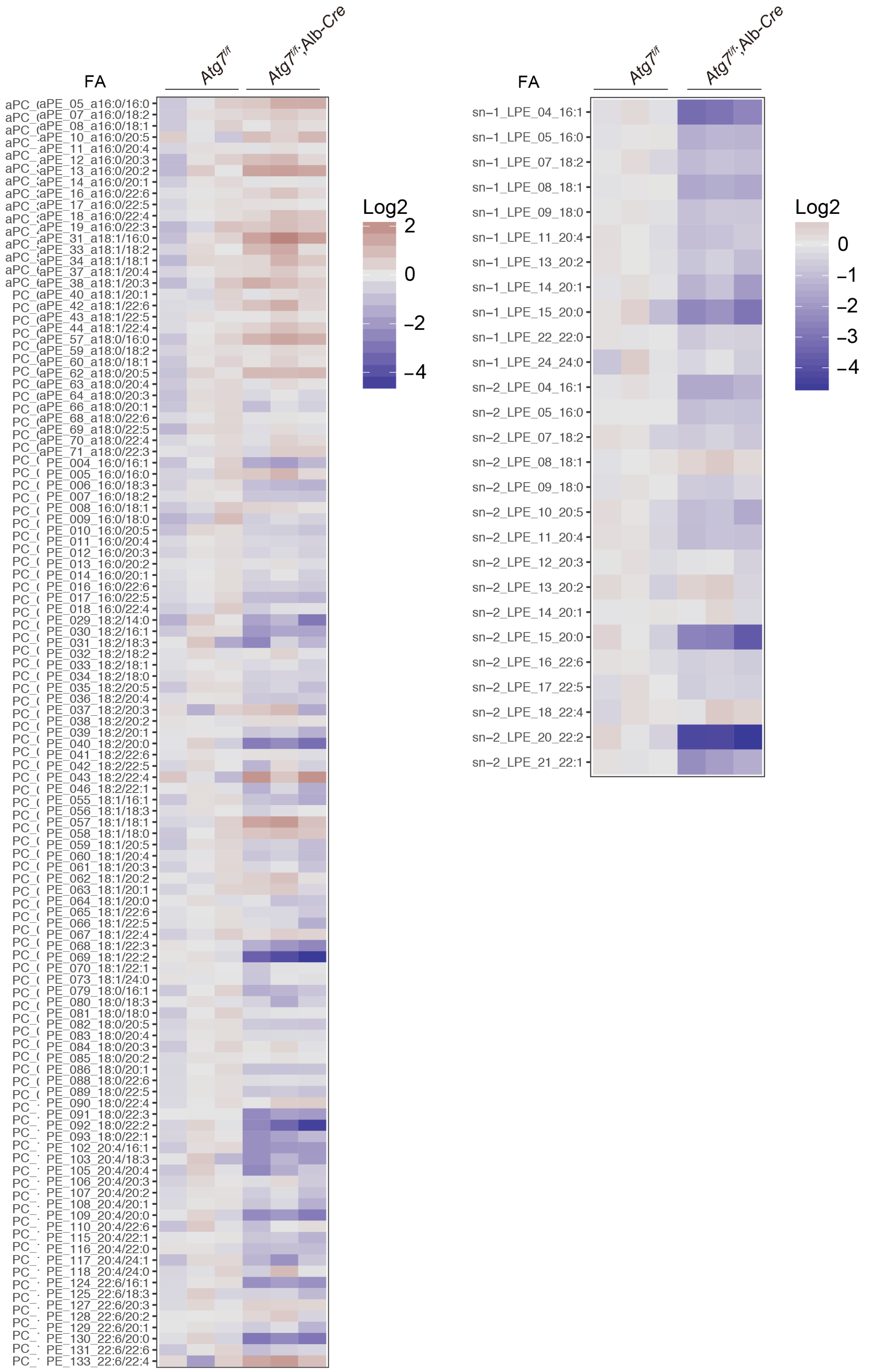
FA
Atg7f/f
Atg7f/f;Alb-Cre
Log2
2
0
−2
−4
sn−1_LPE_04_16:1
sn−1_LPE_05_16:0
sn−1_LPE_07_18:2
sn−1_LPE_08_18:1
sn−1_LPE_09_18:0
sn−1_LPE_11_20:4
sn−1_LPE_13_20:2
sn−1_LPE_14_20:1
sn−1_LPE_15_20:0
sn−1_LPE_22_22:0
sn−1_LPE_24_24:0
sn−2_LPE_04_16:1
sn−2_LPE_05_16:0
sn−2_LPE_07_18:2
sn−2_LPE_08_18:1
sn−2_LPE_09_18:0
sn−2_LPE_10_20:5
sn−2_LPE_11_20:4
sn−2_LPE_12_20:3
sn−2_LPE_13_20:2
sn−2_LPE_14_20:1
sn−2_LPE_15_20:0
sn−2_LPE_16_22:6
sn−2_LPE_17_22:5
sn−2_LPE_18_22:4
sn−2_LPE_20_22:2
sn−2_LPE_21_22:1
−1
−3

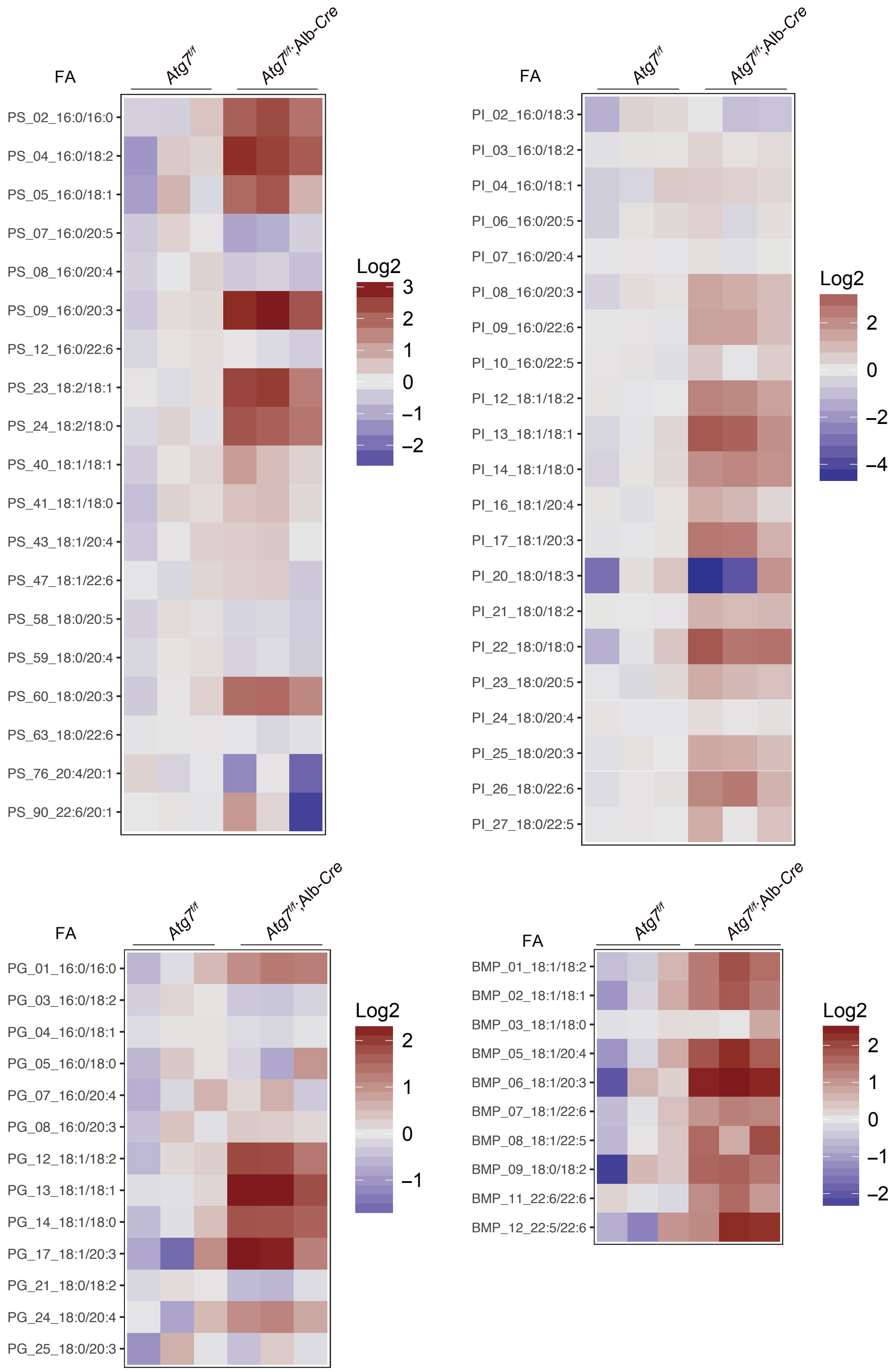
FA
Atg7f/f
Atg7f/f;Alb-Cre
PS_02_16:0/16:0
PS_04_16:0/18:2
PS_05_16:0/18:1
PS_07_16:0/20:5
PS_08_16:0/20:4
PS_09_16:0/20:3
PS_12_16:0/22:6
PS_23_18:2/18:1
PS_24_18:2/18:0
PS_40_18:1/18:1
PS_41_18:1/18:0
PS_43_18:1/20:4
PS_47_18:1/22:6
PS_58_18:0/20:5
PS_59_18:0/20:4
PS_60_18:0/20:3
PS_63_18:0/22:6
PS_76_20:4/20:1
PS_90_22:6/20:1
Log2
3
2
1
0
−1
−2
FA
Atg7f/f
Atg7f/f;Alb-Cre
PI_02_16:0/18:3
PI_03_16:0/18:2
PI_04_16:0/18:1
PI_06_16:0/20:5
PI_07_16:0/20:4
PI_08_16:0/20:3
PI_09_16:0/22:6
PI_10_16:0/22:5
PI_12_18:1/18:2
PI_13_18:1/18:1
PI_14_18:1/18:0
PI_16_18:1/20:4
PI_17_18:1/20:3
PI_20_18:0/18:3
PI_21_18:0/18:2
PI_22_18:0/18:0
PI_23_18:0/20:5
PI_24_18:0/20:4
PI_25_18:0/20:3
PI_26_18:0/22:6
PI_27_18:0/22:5
Log2
2
0
−2
−4
FA
Atg7f/f
Atg7f/f;Alb-Cre
PG_01_16:0/16:0
PG_03_16:0/18:2
PG_04_16:0/18:1
PG_05_16:0/18:0
PG_07_16:0/20:4
PG_08_16:0/20:3
PG_12_18:1/18:2
PG_13_18:1/18:1
PG_14_18:1/18:0
PG_17_18:1/20:3
PG_21_18:0/18:2
PG_24_18:0/20:4
PG_25_18:0/20:3
Log2
2
1
0
−1
FA
Atg7f/f
Atg7f/f;Alb-Cre
BMP_01_18:1/18:2
BMP_02_18:1/18:1
BMP_03_18:1/18:0
BMP_05_18:1/20:4
BMP_06_18:1/20:3
BMP_07_18:1/22:6
BMP_08_18:1/22:5
BMP_09_18:0/18:2
BMP_11_22:6/22:6
BMP_12_22:5/22:6
Log2
2
1
0
−1
−2

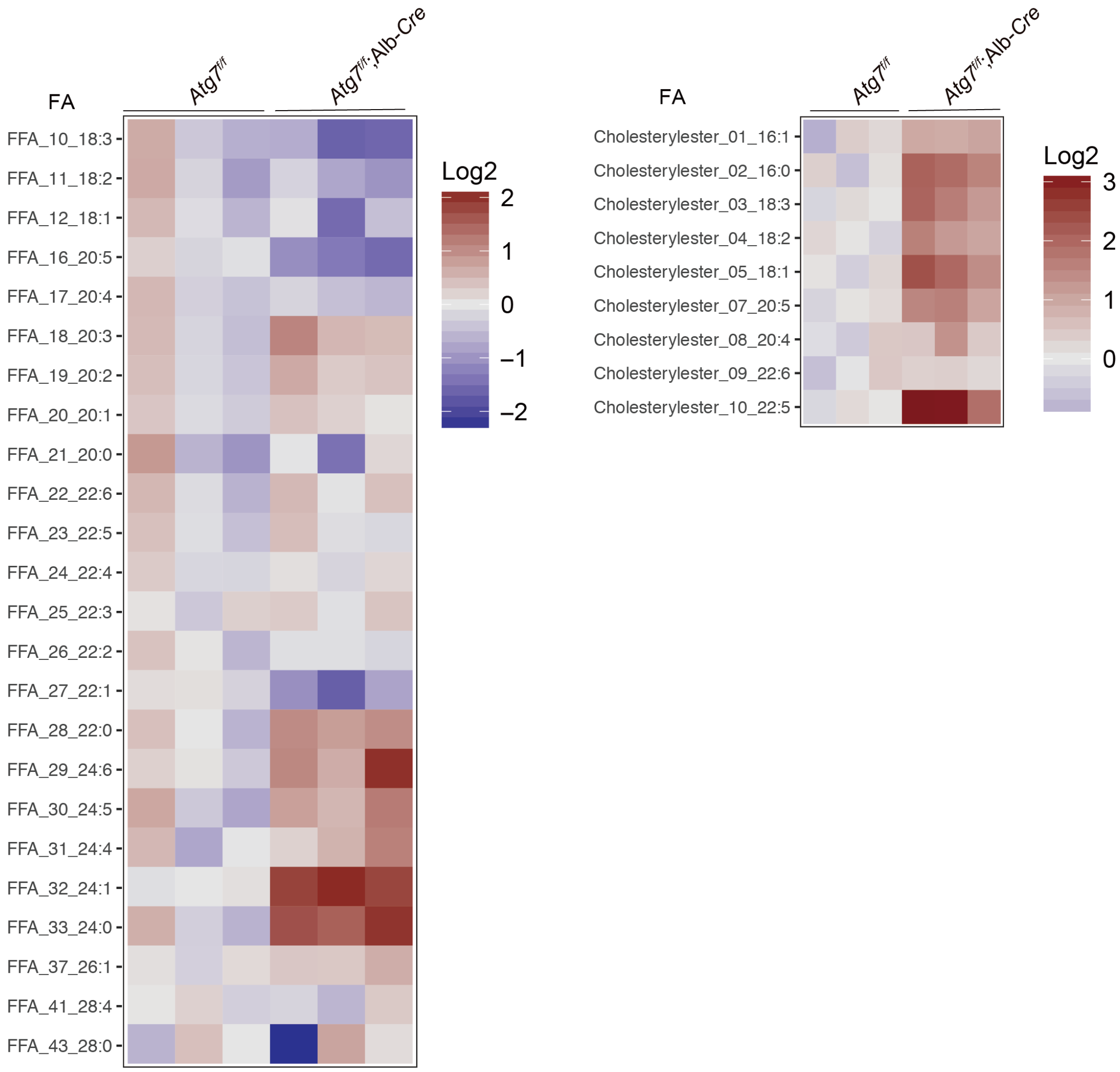

Supplementary Figure 1 Lipidomics of *Atg7*-deficient livers.
Heat maps of 524 lipids in control or *Atg7*-deficient mouse livers (n = 3).

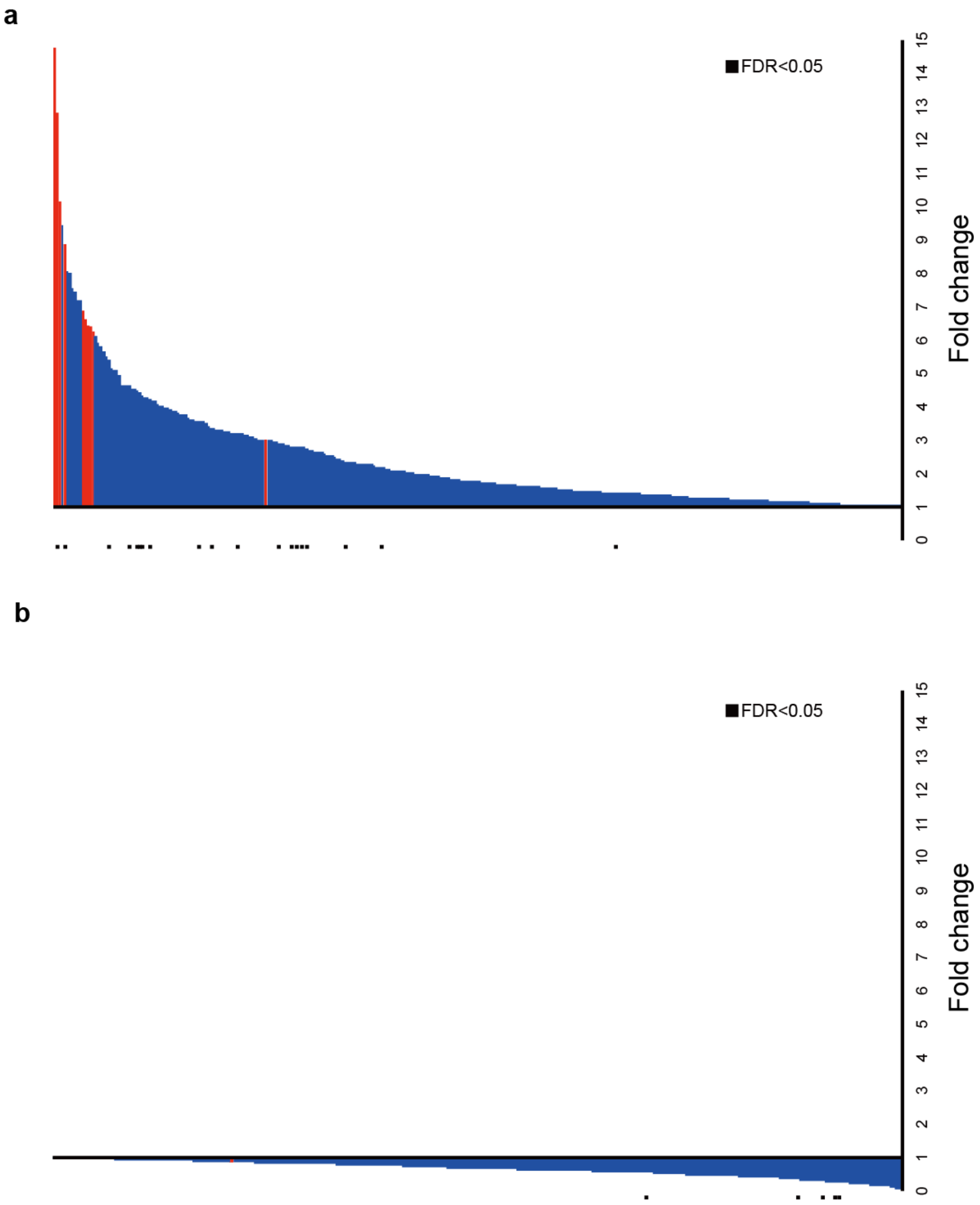

Supplementary Figure 2 Fold change in metabolite concentrations in *Atg7*-deficient livers versus controls.

Fold changes ≥1.0 and <1.0 are visualized in panels a and b, respectively. Red and blue bars indicate acylcarnitine and other metabolites, respectively. *P*-values (Student's *t*-test, two-sided, unequal variance) were corrected by false discovery rate. Black boxes indicate $P < 0.05$.

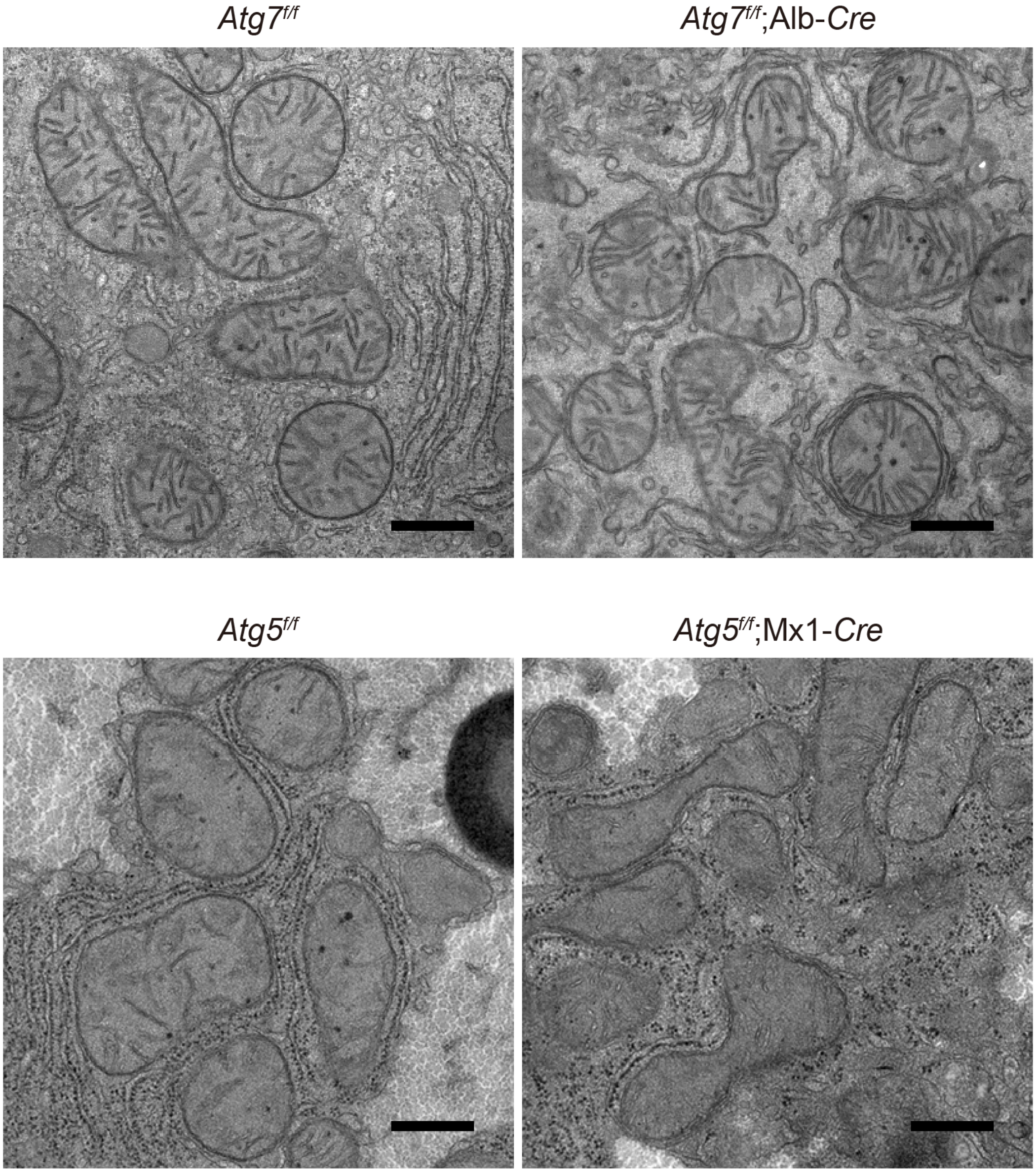

Supplementary Figure 3 Mitochondrial morphology of *Atg7*- and *Atg5*-knockout hepatocytes. Representative electron microscopic images of hepatocytes of the indicated genotype: 5-week-old *Atg7*$^{f/f}$;Alb-*Cre* and the corresponding age-matched controls, *Atg5*$^{f/f}$ and *Atg5*$^{f/f}$;Mx1-*Cre* mice aged 12 weeks. *Atg5*$^{f/f}$ and *Atg5*$^{f/f}$;Mx1-*Cre* mice were intraperitoneally injected with pIpC to delete Atg5 in the liver at the age of 10 weeks. Bars: 500 nm.

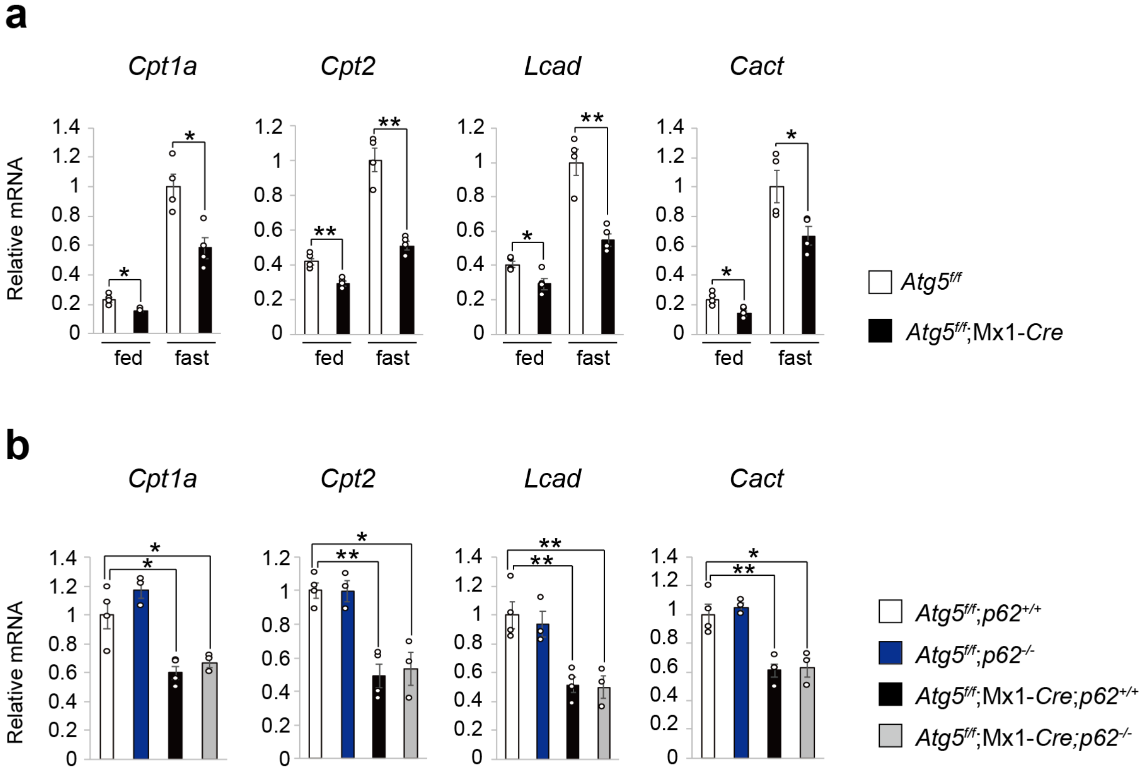

Supplementary Figure 4 Expression of genes encoding enzymes related to lipid oxidation, in *Atg5*- and *Atg5 p62*-double deficient livers.

(**a**) Expression of genes encoding enzymes related to lipid oxidation, in *Atg5*-deficient livers. Total RNAs were prepared from livers of *Atg5*$^{f/f}$ (n = 4) and *Atg5*$^{f/f}$;Mx1-*Cre* (n = 4) mice aged 12 weeks under both fed and fasting conditions. *Atg5*$^{f/f}$ and *Atg5*$^{f/f}$;Mx1-*Cre* mice were intraperitoneally injected with pIpC to delete Atg5 in the liver at the age of 10 weeks. Values were normalized against the amount of mRNA in the liver of pIpC-injected *Atg5*$^{f/f}$ mice. Experiments were performed three times. (**b**) Expression of genes encoding enzymes related to lipid oxidation, in *Atg5 p62*-double deficient livers. Total RNAs were prepared from livers of *Atg5*$^{f/f}$ (n = 4), *Atg5*$^{f/f}$;*p62*$^{-/-}$ (n = 3), *Atg5*$^{f/f}$;Mx1-*Cre*;*p62*$^{+/+}$ (n = 4) and *Atg5*$^{f/f}$;Mx1-*Cre*;*p62*$^{-/-}$ (n = 3) mice aged 12 weeks. *Atg5*$^{f/f}$, *Atg5*$^{f/f}$;*p62*$^{-/-}$, *Atg5*$^{f/f}$;Mx1-*Cre*;*p62*$^{+/+}$ and *Atg5*$^{f/f}$;Mx1-*Cre*;*p62*$^{-/-}$ mice were intraperitoneally injected with pIpC to delete Atg5 in the liver at the age of 10 weeks. Values were normalized against the amount of mRNA in the liver of pIpC-injected *Atg5*$^{f/f}$ mice. Experiments were performed three times. Data are means ± s.e.m. *$P < 0.05$ and **$P < 0.01$ as determined by Welch's *t*-test.

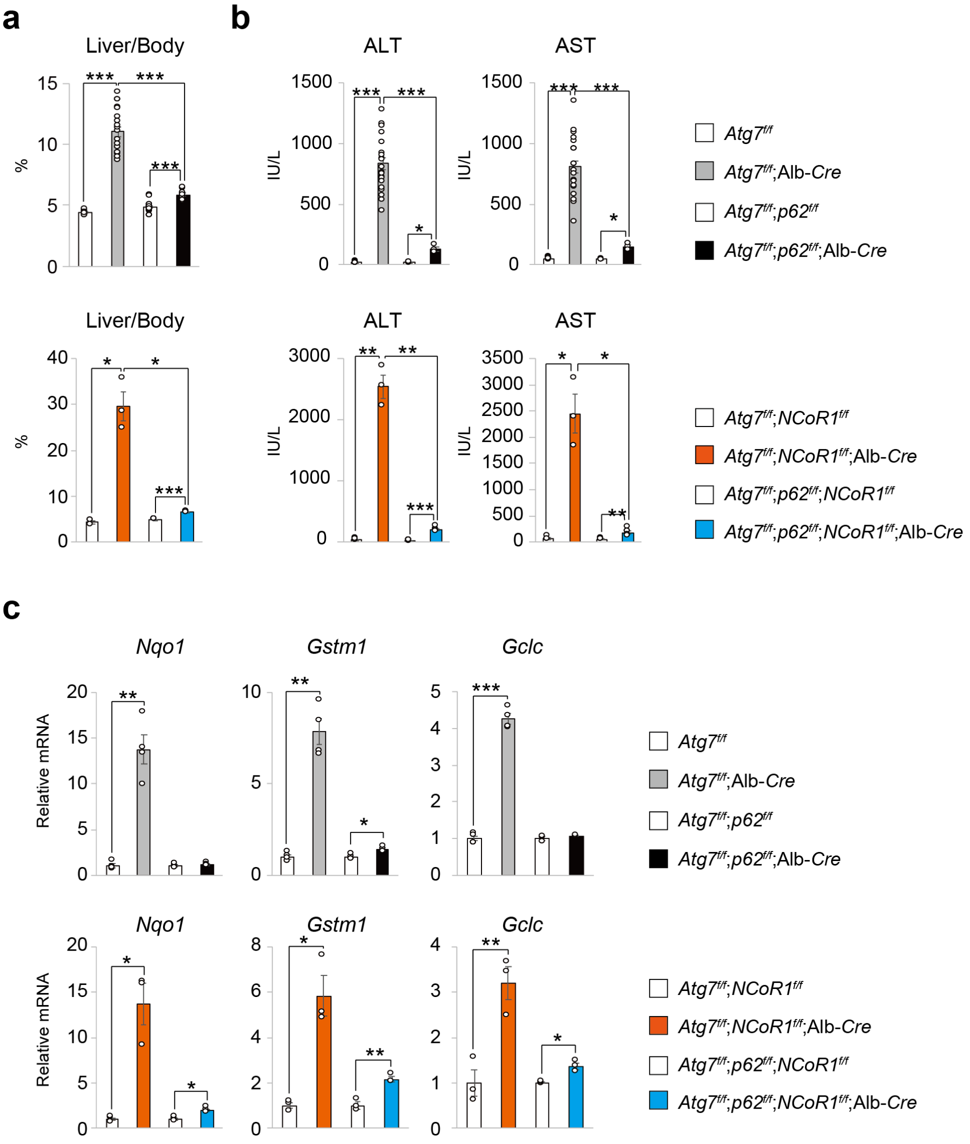

Supplementary Figure 5 Phenotypes of liver-specific *Atg7*-, *Atg7 p62*-, *Atg7 NCoR1*- and *Atg7 p62 NCoR1*-knockout mice.

(**a**) Liver weights (% per body weight) of mice of the indicated genotype: 5-week-old *Atg7*$^{f/f}$;Alb-*Cre* (n = 19) and their corresponding age-matched controls, *Atg7*$^{f/f}$ (n = 7); 5-week-old *Atg7*$^{f/f}$;*p62*$^{f/f}$;Alb-*Cre* (n = 3) and their corresponding age-matched controls, *Atg7*$^{f/f}$;*p62*$^{f/f}$ (n = 3); 12-week-old *Atg7*$^{f/f}$;*NCoR1*$^{f/f}$;Alb-*Cre* (n = 3) and their corresponding age-matched controls, *Atg7*$^{f/f}$;*NCoR1*$^{f/f}$ (n = 3); and 5-week-old *Atg7*$^{f/f}$;*p62*$^{f/f}$;*NCoR1*$^{f/f}$;Alb-*Cre* (n = 5), and their corresponding age-matched controls, *Atg7*$^{f/f}$;*p62*$^{f/f}$;*NCoR1*$^{f/f}$ (n = 5). (**b**) Liver function tests of the mice described in (A). Serum levels of ALT and AST were measured. (**c**) Gene expression of Nrf2 target genes in livers of 5-week-old *Atg7*$^{f/f}$;Alb-*Cre* (n = 4) and their corresponding age-matched controls, *Atg7*$^{f/f}$ (n = 4); 5-week-old *Atg7*$^{f/f}$;*p62*$^{f/f}$;Alb-*Cre* (n = 3) and their corresponding age-matched controls, *Atg7*$^{f/f}$;*p62*$^{f/f}$ (n = 3); 12-week-old *Atg7*$^{f/f}$;*NCoR1*$^{f/f}$;Alb-*Cre* (n = 3) and their corresponding age-matched controls, *Atg7*$^{f/f}$;*NCoR1*$^{f/f}$ (n = 3); and 5-week-old *Atg7*$^{f/f}$;*p62*$^{f/f}$;*NCoR1*$^{f/f}$;Alb-*Cre* (n = 4) and their corresponding age-matched controls, *Atg7*$^{f/f}$;*p62*$^{f/f}$;*NCoR1*$^{f/f}$ (n = 4). Values were normalized against the amount of mRNA in the livers of control mice. Experiments were performed three times. Data are means ± s.e.m. $*P < 0.05$, $**P < 0.01$, and $***P < 0.001$ as determined by Welch's *t*-test.

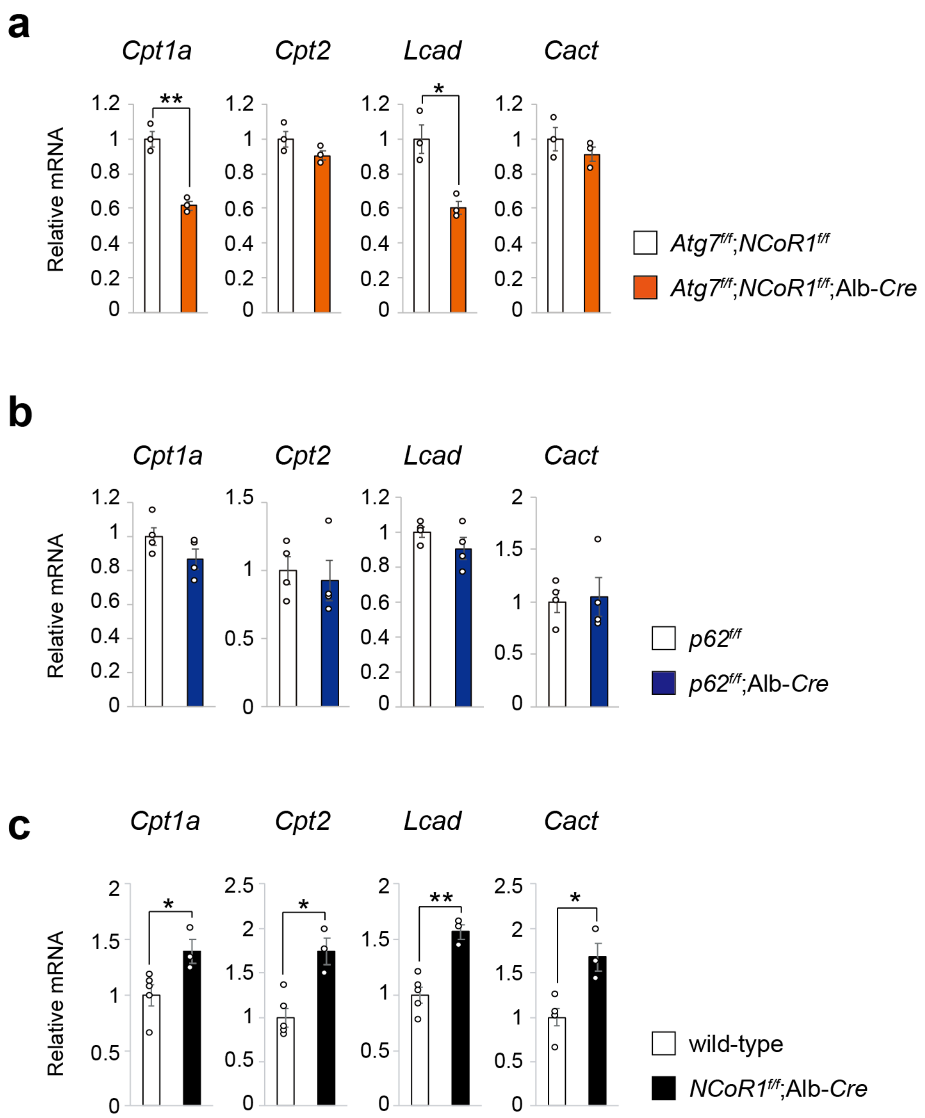

Supplementary Figure 6 Gene expression of enzymes related to lipid oxidation in *Atg7 NCoR1* double-knockout, *p62*-knockout and *NCoR1*-knockout livers.

(**a**–**c**) Gene expression of enzymes related to lipid oxidation in *Atg7 NCoR1* double-knockout (a), *p62*-knockout (b) and *NCoR1*-knockout (c) livers. Total RNAs were prepared from livers of 12-week-old *Atg7*$^{f/f}$;*NCoR1*$^{f/f}$;Alb-*Cre* (n = 3) and their corresponding age-matched controls, *Atg7*$^{f/f}$;*NCoR1*$^{f/f}$ (n = 3) mice; 5-week-old *p62*$^{f/f}$;Alb-*Cre* (n = 4) and their corresponding age-matched controls, *p62*$^{f/f}$ (n = 4); and 10-week-old *NCoR1*$^{f/f}$;Alb-*Cre* (n = 3) and age-matched wild-type mice (n = 5). Values were normalized against the amount of mRNA in the livers of control mice. Experiments were performed three times. Data are means ± s.e.m. *$P < 0.05$, and **$P < 0.01$ as determined by Welch's *t*-test.

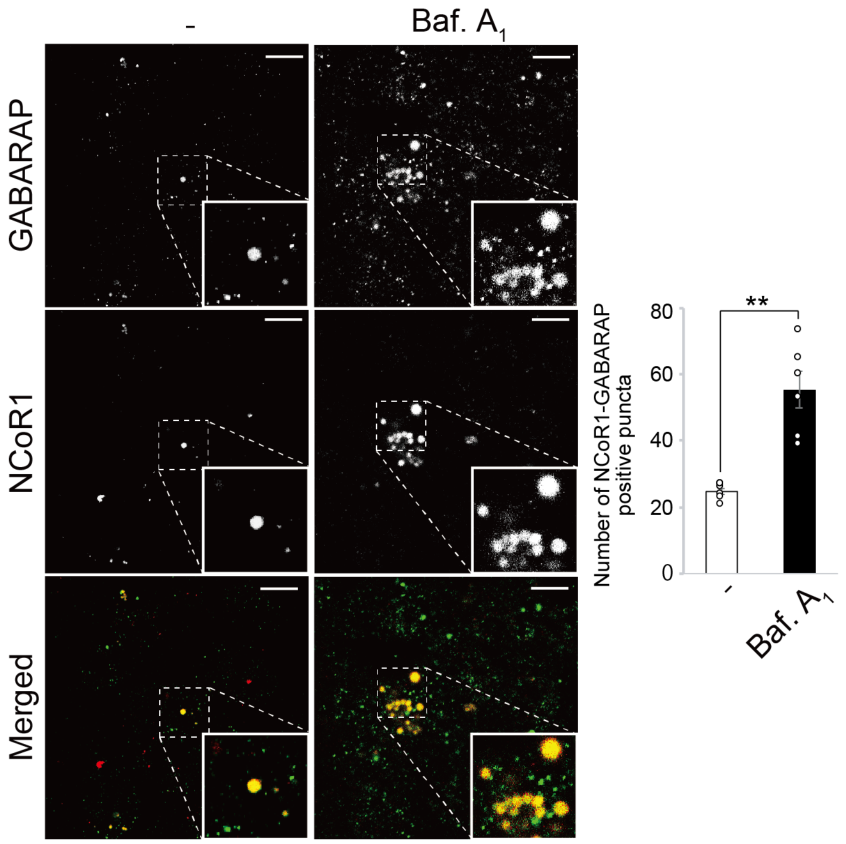

Supplementary Figure 7 Colocalization of NCoR1 with GABARAP-positive structures. Immunofluorescence analysis. Wild-type or *ATG7*-deficient HepG2 cells were cultured in the presence or absence of bafilomycin A_1 (Baf A_1) for 24 hr., and then immunostained with anti-GABARAP and anti-NCoR1 antibodies. The number of cytoplasmic NCoR1 and GABARAP double-positive dots per 20 cells was counted. The experiments were performed three times. Data are means ± s.e.m. **P* < 0.05 as determined by Welch's *t*-test. Each inset is a magnified image. Bar: 2.5 μm.

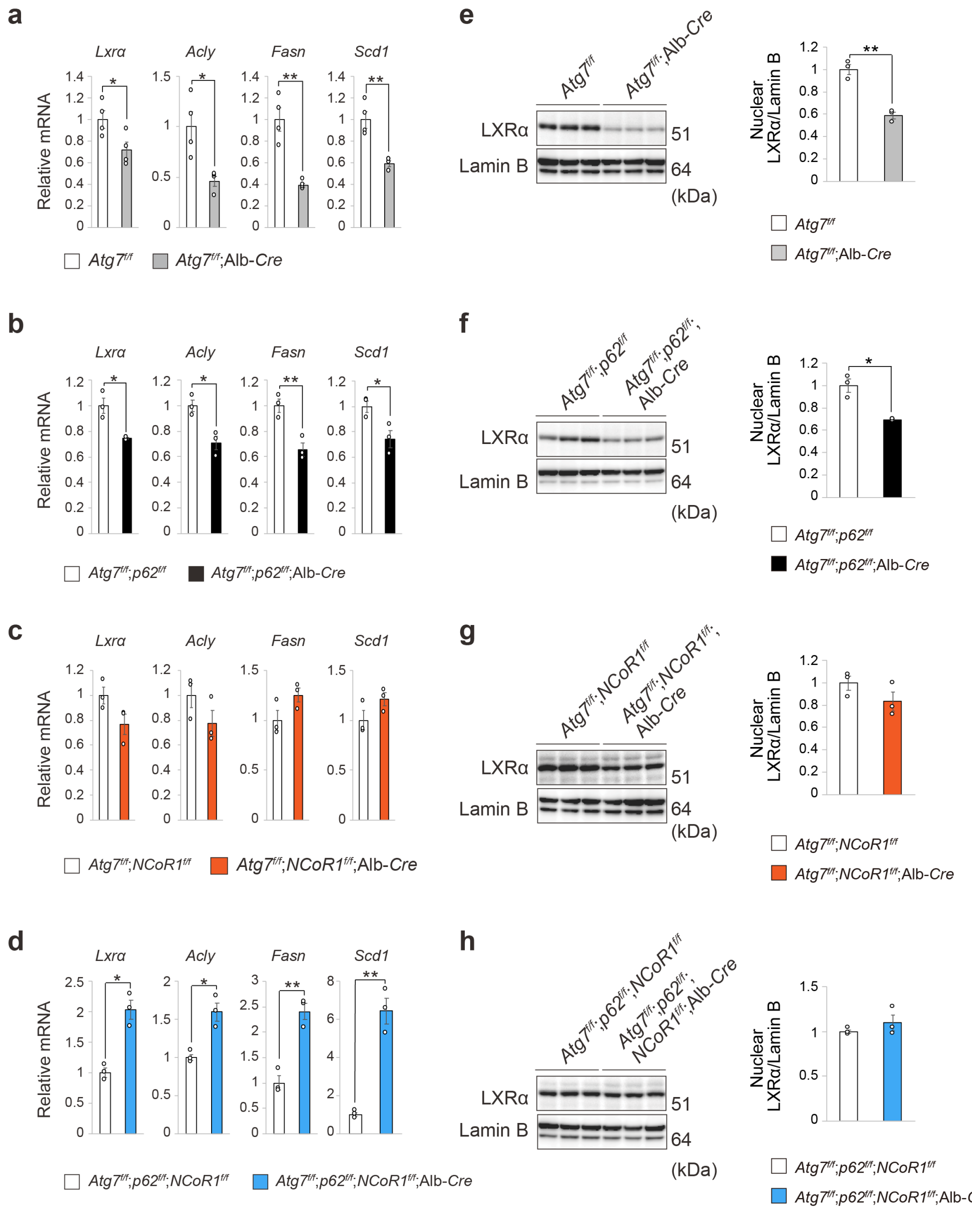

Supplementary Figure 8 NCoR1-dependent inactivation of LXRα in *Atg7*-deficient livers. (**a**–**d**) Expression of LXRα target genes in the livers of *Atg7*-knockout (a), *Atg7 p62* double-knockout (b), *Atg7 NCoR1* double-knockout (c) and *Atg7 p62 NCoR1* triple-knockout (d) mice. Total RNAs were prepared from livers of 5-week-old *Atg7*$^{f/f}$;Alb-*Cre* (n = 4) and their corresponding age-matched controls, *Atg7*$^{f/f}$ (n = 4); 5-week-old *Atg7*$^{f/f}$;*p62*$^{f/f}$;Alb-*Cre* (n = 4) and their corresponding age-matched controls, *Atg7*$^{f/f}$;*p62*$^{f/f}$ (n = 4), 12-week-old *Atg7*$^{f/f}$;*NCoR1*$^{f/f}$;Alb-*Cre* (n = 3) and their corresponding age-matched controls, *Atg7*$^{f/f}$;*NCoR1*$^{f/f}$ (n = 3); and 5-week-old *Atg7*$^{f/f}$;*p62*$^{f/f}$;*NCoR1*$^{f/f}$;Alb-*Cre* (n = 4) and their corresponding age-matched controls, *Atg7*$^{f/f}$;*p62*$^{f/f}$;*NCoR1*$^{f/f}$ (n = 4). Values were normalized against the amount of mRNA in the livers of control mice. Experiments were performed three times. (**e**–**h**) LXRα level in the livers of *Atg7*-knockout (e), *Atg7 p62* double-knockout (f), *Atg7 NCoR1* double-knockout (g) and *Atg7 p62 NCoR1* triple-knockout (h) mice. Nuclear fraction was prepared from livers of mice of the indicated genotypes and subjected to immunoblotting with the indicated antibodies. Bar graphs indicate the quantitative densitometric analyses of nuclear LXRα relative to Lamin B. Data are means ± s.e.m. *$P < 0.05$, and **$P < 0.01$ as determined by Welch's *t*-test.

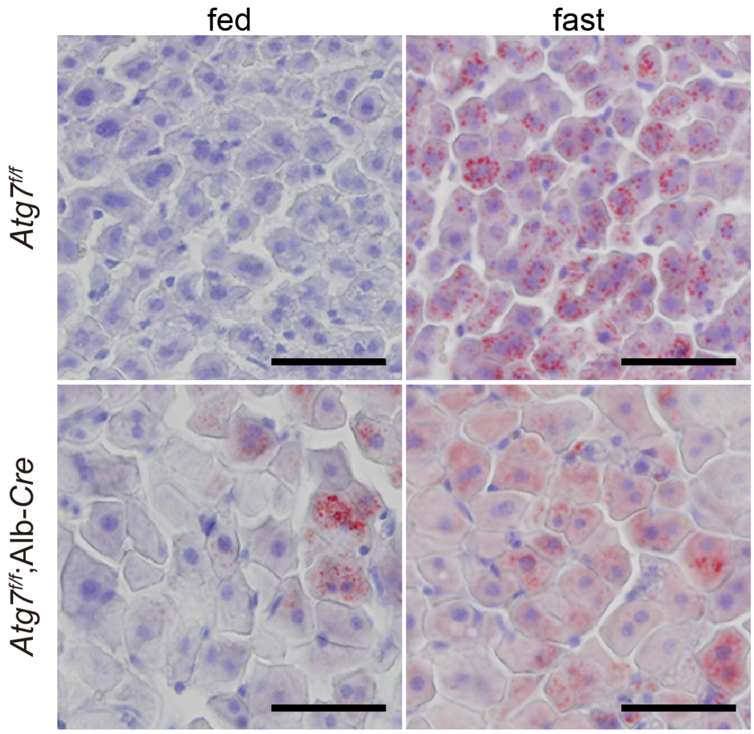

Supplementary Figure 9 Suppression of hepatosteatosis in response to fasting in *Atg7*-knockout livers.

Oil-red staining. Liver sections of *Atg7*$^{f/f}$ and *Atg7*$^{f/f}$;Alb-*Cre* mice aged 5 weeks were stained with oil red O. Bars: 50 μm.

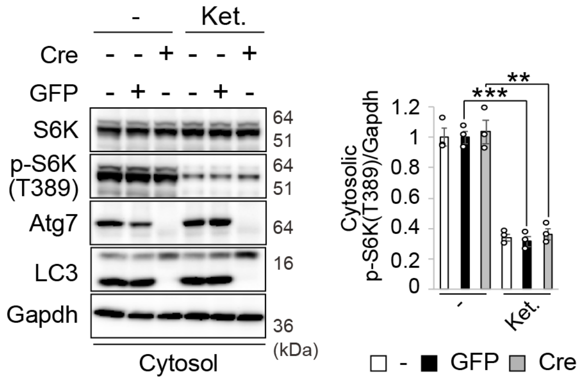

Supplementary Figure 10 Phosphorylation level of RPS6KB1 (S6K) in *Atg7*-deficient hepatocytes.

Primary hepatocytes were isolated from *Atg7*$^{f/f}$ mice and infected with adenovirus for GFP or Cre recombinase. Forty-eight hours after infection, the cells were cultured under nutrient-rich and ketgenic conditions for 24 hr, and then cytoplasmic fraction was prepared from the cells and subjected to immunoblotting with the indicated antibodies. Data are representative of three separate experiments. Bar graphs indicate the quantitative densitometric analyses of cytoplasmic phosphorylated S6K relative to Gapdh. Data are means ± s.e.m. **P < 0.01, and ***P < 0.001 as determined by Welch's t-test.

Full blot images for Figure 2b

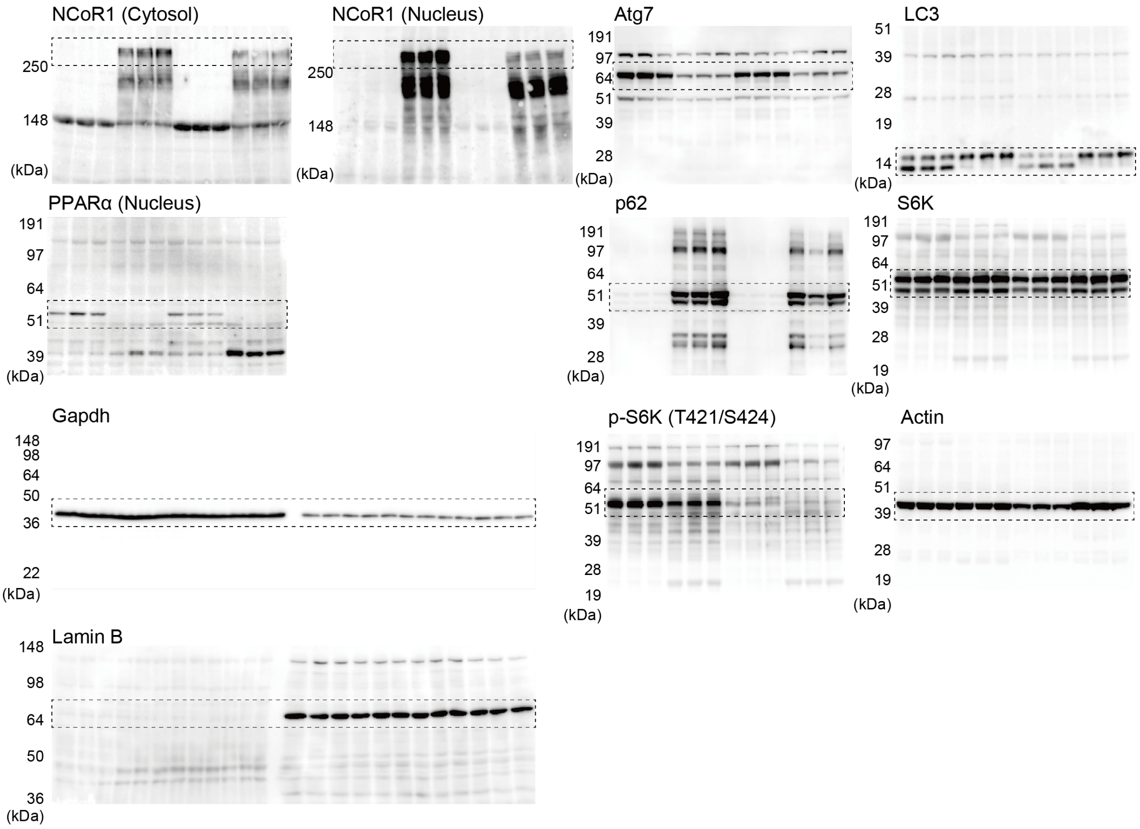

Full blot images for Figure 2c

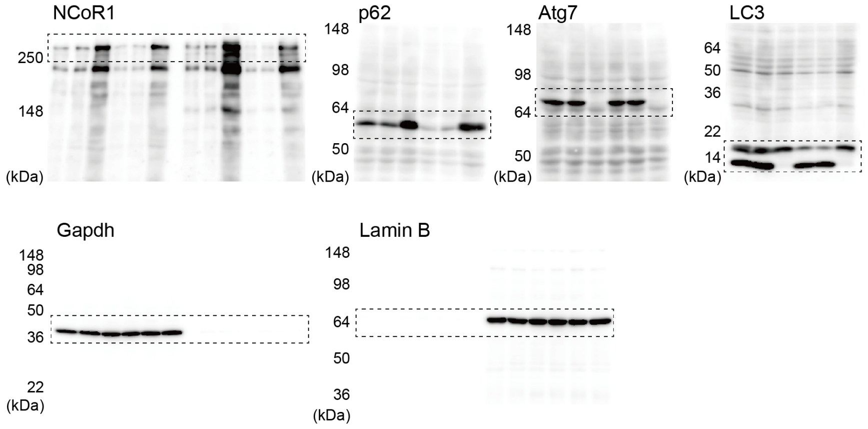

Supplementary Figure 11 Full blot images for Figures 2b and 2c.

Full blot images for Figure 3a

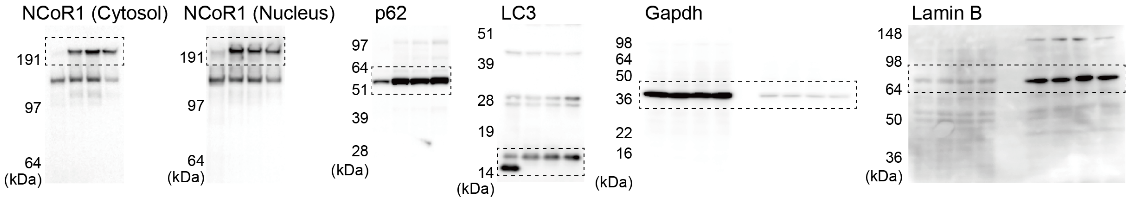

Full blot images for Figure 4a

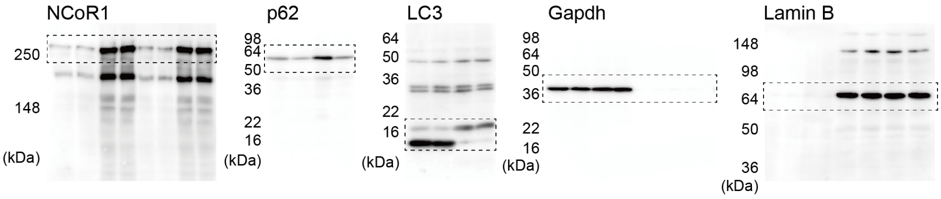

Full blot images for Figure 4c

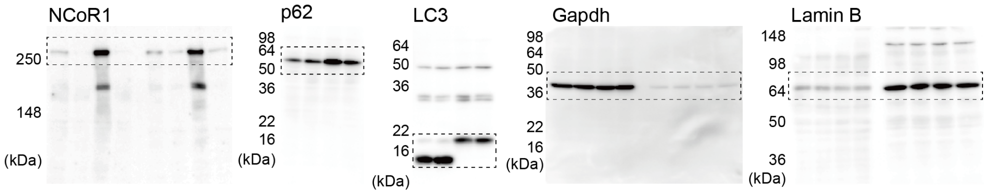

Full blot images for Figure 4e

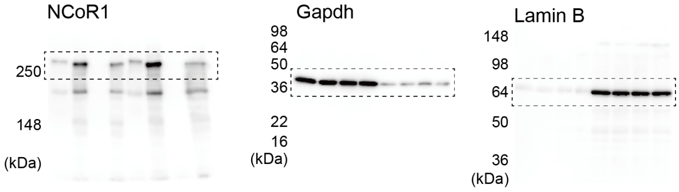

Supplementary Figure 12 Full blot images for Figures 3a, 4a, 4c and 4e.

Full blot images for Figure 5b

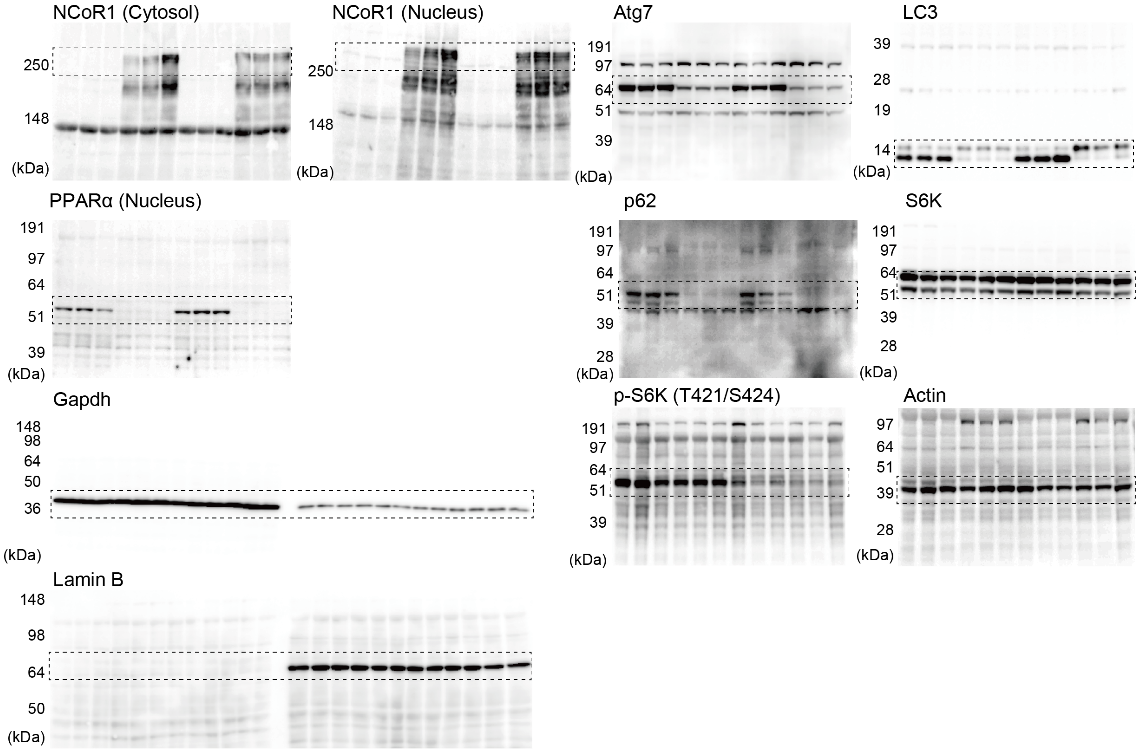

Full blot images for Figure 5d

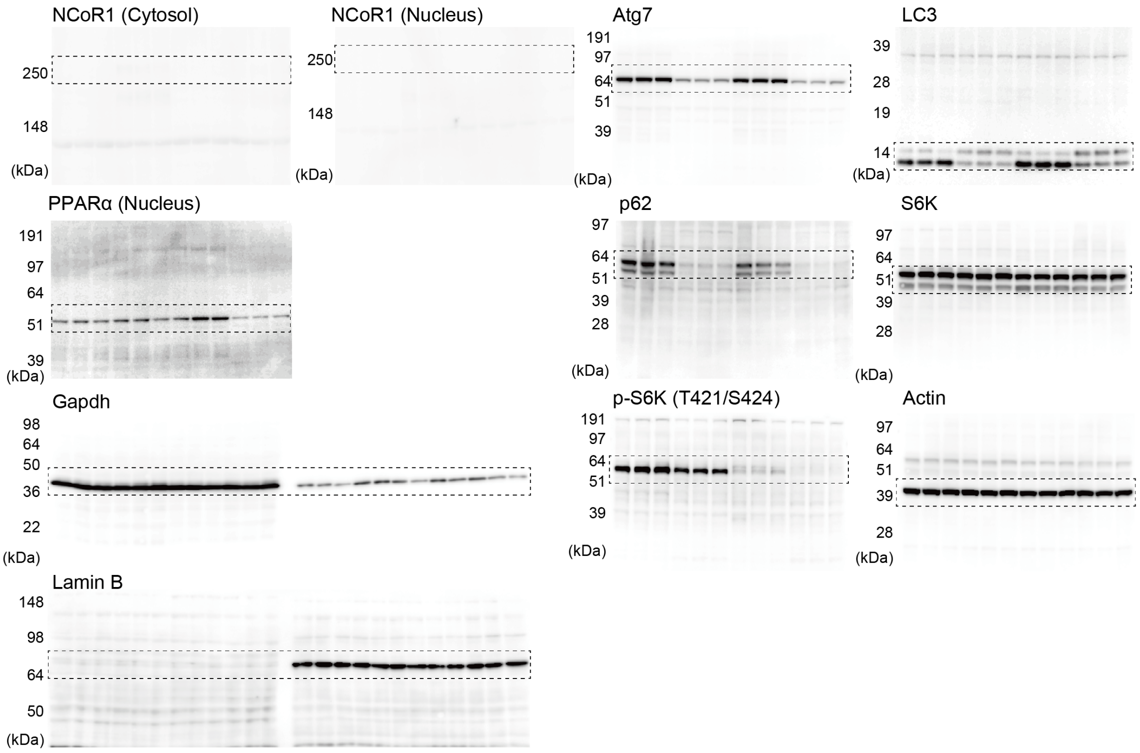

Supplementary Figure 13 Full blot images for Figures 5b and 5d.

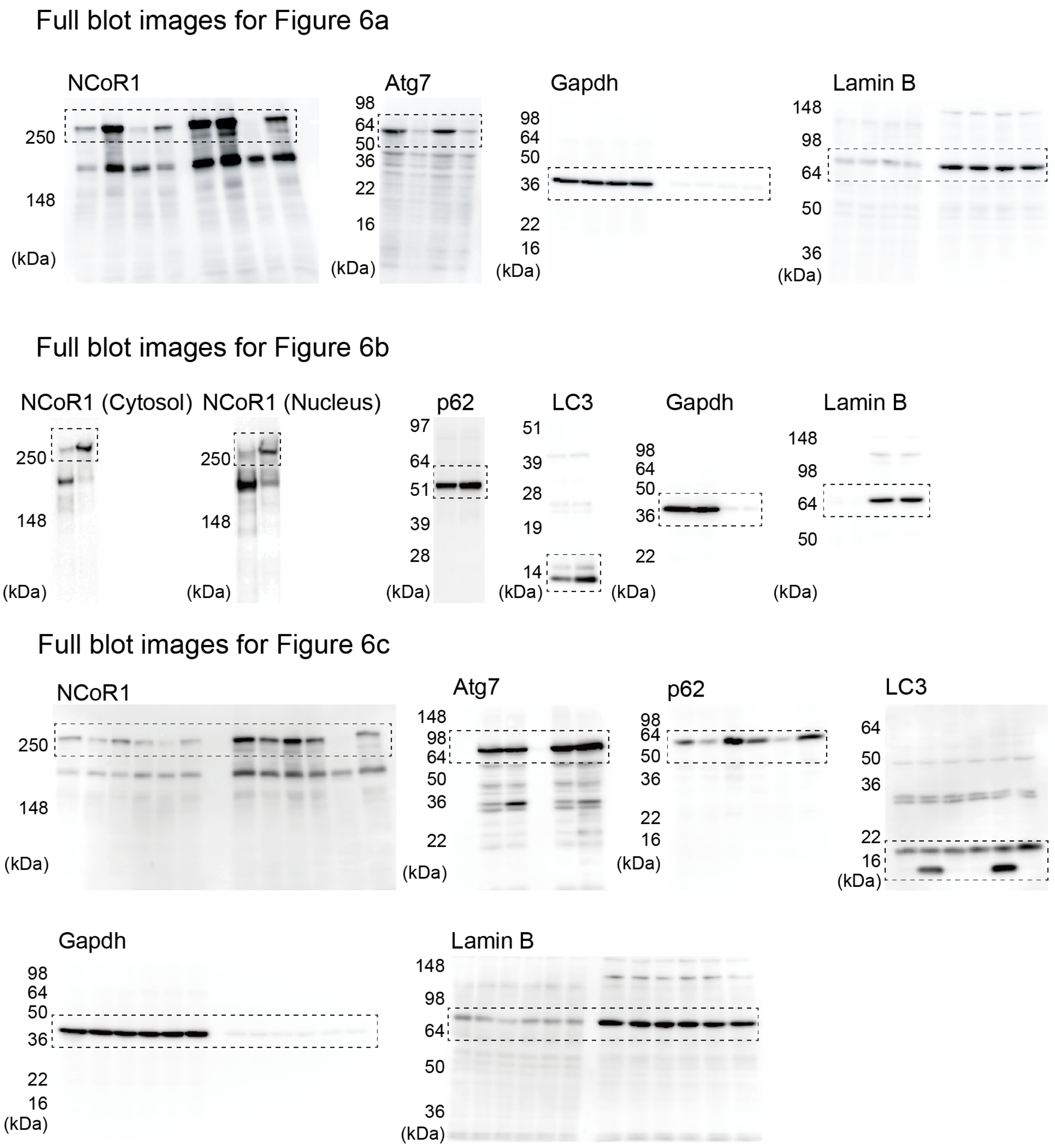

Supplementary Figure 14 Full blot images for Figures 6a, 6b and 6c.

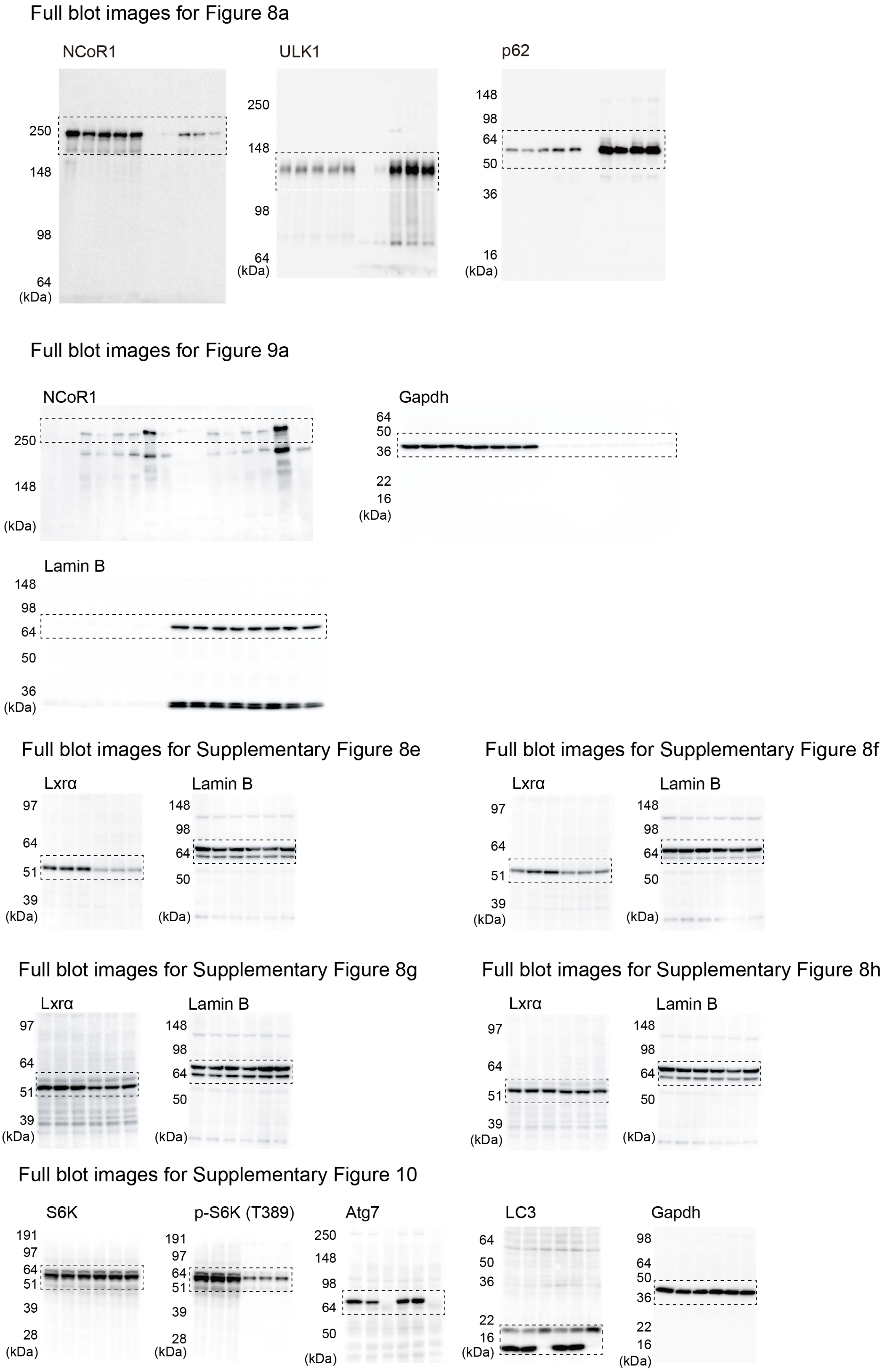

Supplementary Figure 15 Full blot images for Figures 8a, 9a, Supplementary Figure 8e, 8f, 8g, 8h and 10.

Supplementary Table 1
Primer sequences used in quantitative real-time PCR.

Gene	Left	Right
MmGus	ctctggtggccttacctga	ctcagttgttgtcaccttcacc
MmCpt1a	gactccgctcgctcattc	tctgccatcttgagtggtga
MmCpt2	ccaaagaagcagcgatgg	tagagctcaggcagggtga
MmLcad	aagtgattcctcaccacacaga	cagctttttcccagacctctc
MmCact	aaatctccagaggatgaactta	cctgtggtgaacacaccagata
MmPparα	aactggatgacagtgacatttcc	ccctcctgcaacttctcaat
MmNCoR1	ttctgaaattattgatggtctttctg	acagaaagctgacgcatttg
MmLxrα	gagtgtcgacttcgcaaatg	cggatctgttcttctgacagc
MmAcly	gtggccccaactatcaagag	atggggatcccagtggtc
MmFasn	gctgctgttggaagtcagc	agtgttcgttcctcggagtg
MmScd1	ttccctcctgcaagctctac	cagagcgctggtcatgtagt
HsGAPDH	acgggaagcttgtcatcaat	catcgccccacttgatttt
HsCPT1A	caatcggactctggaaacg	ccgctgaccacgttcttc
HsCPT2	tgaccaaagaagcagcaatg	gagctcaggcaagatgatcc
HsHADH	ctcggccaagaagataatcg	tctaccaacactactgtgtgacca
HsNQO1	atcctgccgagtctgttctg	agggactccaaaccactgc
HsGCLC	ggatgatgctaatgagtctgacc	tctactctccatccaatgtctgag
HsUGDH	gtagctcgttattggcagca	atctatgatccgggaagcaa

Supplementary Table 2
Primer sequences used in Chromatin immunoprecipitation (ChIP) coupled with quantitative PCR.

Gene	Left	Right
hsNQO1-ARE	catgtctccccaggactctc	ttttagccttggcacgaaat
hsGATA1-ex3	gcctcaactgtgtgtcccac	gaaggtactggaaaagtcag
hsCPT1A	caccacggctgatttttgta	ccaggagcagtgggatagaa
hsCPT2	gtccacagtctcgcaaggat	ccctaggaggcgggaaac
